# Case report: Rupture of an ileus tube in a patient with recurrent rectal cancer

**DOI:** 10.3389/fonc.2023.1270728

**Published:** 2023-12-15

**Authors:** Jun Ma, Ye Jiang, Chaoping Zhou, Datian Wang, Chunxia Zhao, Yaming Zhang

**Affiliations:** ^1^ Department of General Surgery, Anqing Municipal Hospital, Anqing, China; ^2^ Department of Gastroenterology, Anqing Municipal Hospital, Anqing, China

**Keywords:** rectal cancer, recurrence or metastasis, intestinal obstruction, ileus tube, complication

## Abstract

The insertion of an ileus tube is an important treatment for intestinal obstruction. According to previous reports, jejunal intussusception has been reported as a complication associated with ileus tube placement. However, rupture of the weighted tip of an ileus tube has not been reported before. Herein, we report a 55-year-old Chinese woman who underwent radical proctectomy (DIXON) for rectal cancer and developed pelvic recurrence and lung metastasis 65 months after surgery, accompanied by symptoms of acute intestinal obstruction. An ileus tube was inserted before the operation (extensive total hysterectomy, bilateral adnexal resection, rectal Hartman operation, partial enterectomy, and intestinal adhesion lysis). Rupture of the ileus tube occurred after the operation and was treated with paraffin oil and enteral nutrition, and the metal beads and spring were eliminated through the colostomy. During the follow-up, the patient received targeted therapy plus immunotherapy, which was successful: the quality of life of the patient was excellent, and no obvious abnormal symptoms were found. Endoscopy-assisted ileus tube insertion should be performed under intravenous anesthesia, and a knot should be tied at the tip of the ileus tube before insertion so that the ileus tube can be inserted easily by grasping the thread with biopsy forceps(the “thread-knotting” method). With the above methods, the procedure of ileus tube insertion could be improved to reduce the incidence of tube-related rupture.

## Background

The insertion of an ileus tube is an important treatment for patients with intestinal obstruction. It reduces the rate of surgery, provides effective preoperative preparation for surgery, and decreases the incidence of surgical complications (1–3). In addition, for patients with advanced intestinal tumors, the ileus tube plays an important role in bowel rest and effective decompression,which is a therapeutic method with high efficacy and low invasiveness (4). Compared to a gastric tube, a transnasal ileus tube is more effective at decompression for patients with intestinal obstruction (5). According to previous reports, jejunal intussusception has been reported as a complication associated with ileus tube placement (6–8). However, rupture of the weighted tip of an ileus tube has not been reported before.This study reports a rare rupture of the ileus tube in a patient with recurrent rectal cancer, and discusses the diagnosis and prevention associated with this case.

## Case presentation

A 55-year-old Chinese woman who underwent radical proctectomy because of rectal cancer 65 months prior.On September 20, 2016, the patient underwent radical proctectomy (DIXON) for rectal cancer under general anesthesia. Postoperative pathologic examination showed stage IIIB, moderately differentiated adenocarcinoma with lymph node involvement (5/17) and two tumor deposits in the rectal mesentery. The circumferential resection margin (CRM) was negative (2 mm). The immunohistochemical marker results were as follows: CK (+), HER-2 (-), p53 (+), Ki-67 (approximately 70%), MSH2 (+), MSH6 (+), MLH1 (+), and PMS2 (+). The patient continued adjuvant chemotherapy after surgery, completing eight cycles of oxaliplatin and capecitabine (CAPEOX regimen).

Four years post surgery, CT showed multiple metastatic nodules located in both lungs, and there were malignant soft masses in the uterus and adnexa. On October 8, 2020, MRI showed multiple metastatic soft masses located in the uterus and adnexa, and there were multiple encapsulated effusions in the pelvis. Transcervical biopsy showed metastatic adenocarcinoma of rectal origin. Genetic testing revealed no mutations in the Braf or Ras gene. The patient was treated with FOLFOX6 plus cetuximab for 6 cycles, and the lesion did not progress. However, the patient’s regimen was changed to FOLFIRI plus cetuximab because of numbness in the hands and feet, and 3 cycles of the regimen were completed. Due to the progression of the pelvic and pulmonary lesions, the treatment regimen was changed to XELOX + bevacizumab. Seven cycles of this regimen were completed.

**Figure 1:**
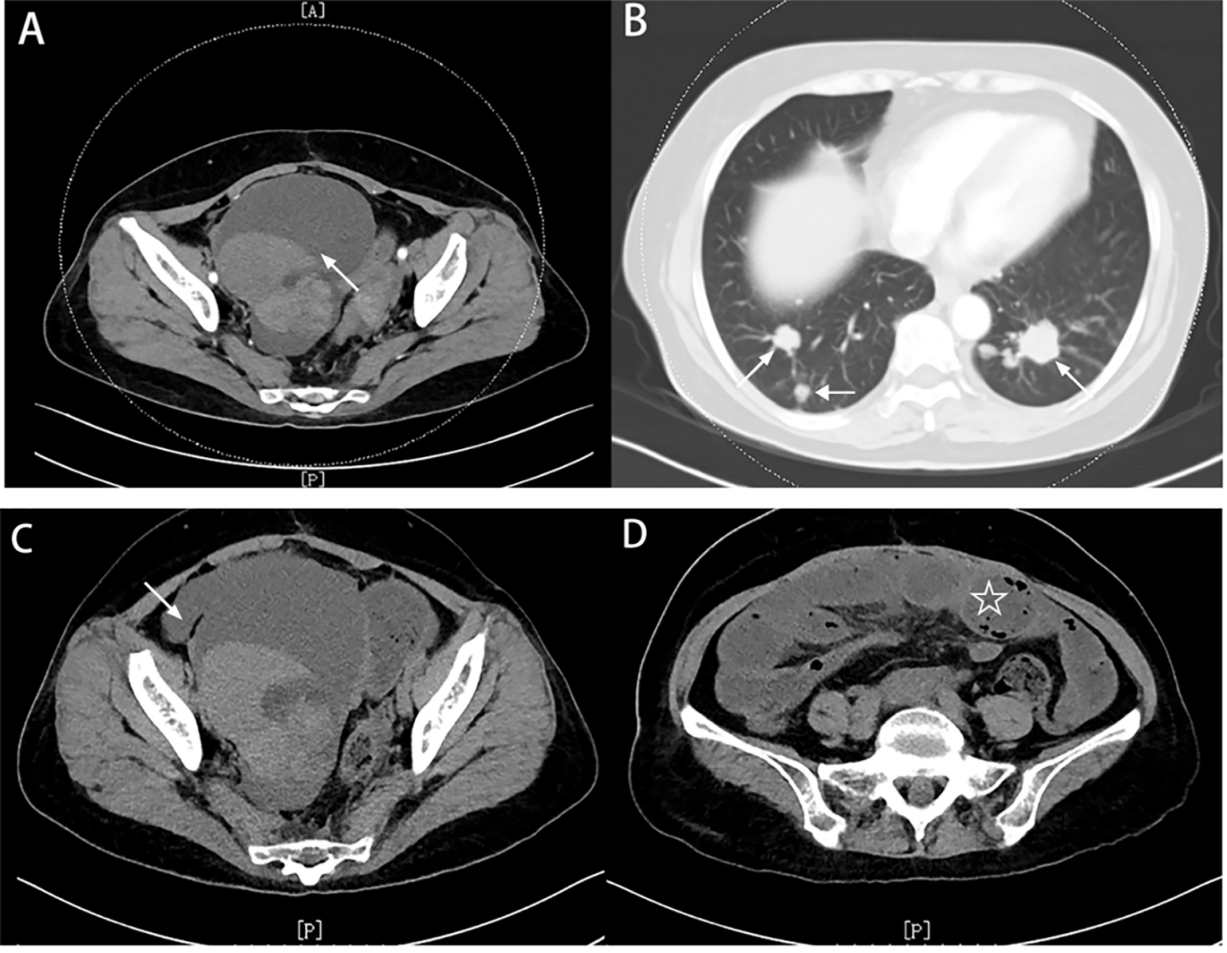
Imaging before the insertion of an ileus tube **(A)** CT showed that the pelvic lesions (white arrow) had progressed significantly. **(B)** CT showed that multiple metastatic nodules (white arrow) were located in both lungs. **(C)** CT showed that the obstruction was located in the terminal ileum that had been invaded by the large pelvic tumor (white arrow). **(D)** The small bowel became significantly dilated due to the obstruction (white asterisk).

In March, 2022, the woman was admitted to the hospital because of sudden pain in the lower right abdomen. She had no chills, fever, vomiting or blood in the stool at the time of admission. The patient had diabetes and hypertension, her blood glucose level was 7.76 mmol/ml, and her CRP level was 33.32 mg/L. Other parameters were normal. CT showed that the lung and pelvic lesions had progressed significantly (**Figures 1A, B**). After a week of oral fruquintinib therapy, the patient had worsening abdominal pain and stopped having farts and defecation. Her abdomen was distended, and a hard mass was palpated in the right lower abdomen with clear borders and fair mobility, and she had tenderness pain, and intestinal sounds in her abdominal cavity. The site of obstruction was located in the terminal ileum invaded by the large pelvic tumor (**Figures 1C, D**).

As the symptoms of intestinal obstruction significantly worsened, the ileus tube (CLINY Ileus tube suite, Create Medic, Tokyo, Japan; 300 cm in length, 16 Fr) was inserted under endoscopic guidance on March 23, 2022. The decompression tube was first placed into the stomach through the nasal cavity, and then the gastroscope was placed into the gastric cavity. After endoscopic suction of the stomach contents, the tube was moved into the descending duodenum by forceps, where it was kept fixed. Then the anterior balloon was inflated with 20 mL distilled water. The gastroscope was withdrawn after the long tube was fixed to the cheek. The tube was propelled by bowel peristalsis and its weighted tip, and the outside terminal of the tube was connected to a spontaneous negative-pressure bag.

**Figure 2:**
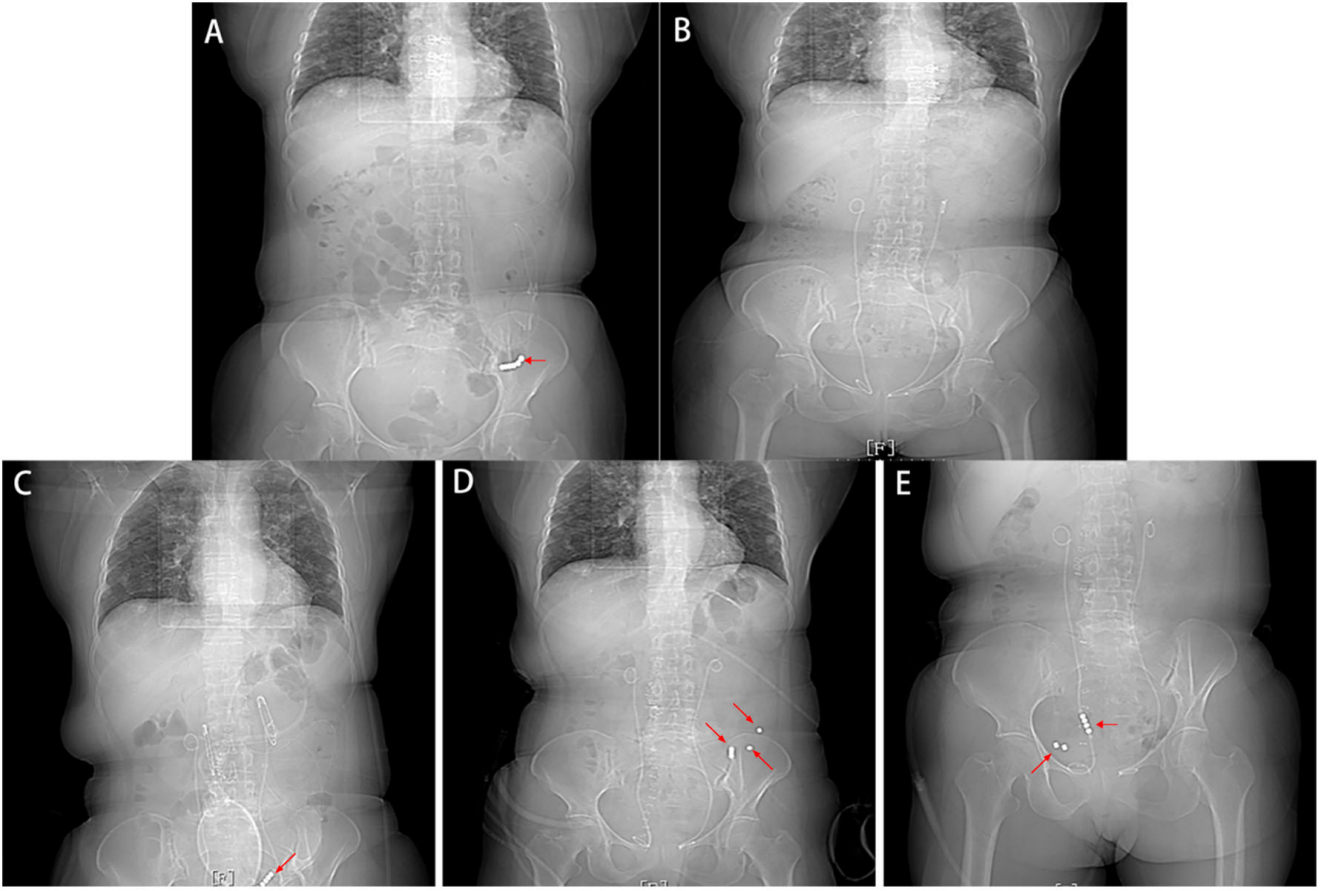
Imaging after the insertion of an ileus tube **(A)** CT showed that the intestinal obstruction was improved, and the head of the tube was now in the left lower abdomen. **(B)** CT showed no metallic shadow, and the rest of the CT findings were normal. **(C)** CT did not show intestinal obstruction or intestinal leakage but showed separation between the metal beads at the head of the ileus tube. **(D, E)** Follow-up CT showed that six scattered metal beads had moved toward the distal small intestine with no signs of intestinal obstruction or perforation.

The patient was ventilated and defecated via decompression. On March 31, 2022, CT showed that the intestinal obstruction had improved, and the head of the tube was now in the left lower abdomen (**Figure 2A**). Since the obstruction was only temporarily relieved and the patient was otherwise still unable to eat and continue treatment, surgery needed to be performed eventually.

Bilateral ureteral catheterization was performed before surgery (to avoid a ureteral injury), and on April 4, 2022, extensive total hysterectomy + bilateral adnexal resection + rectal Hartmann operation + partial small bowel resection + intestinal adhesion release was performed.

**Figure 3:**
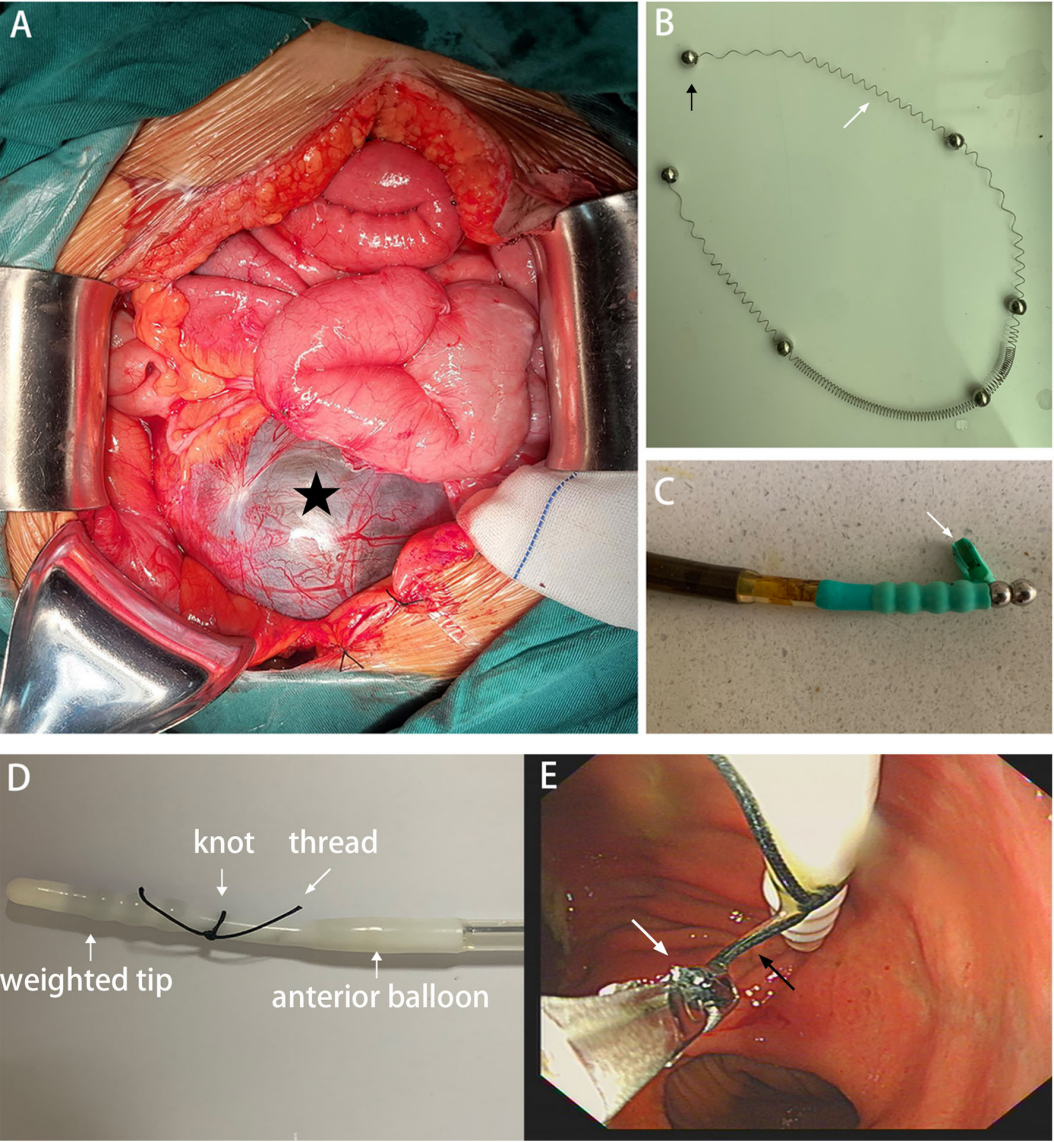
**(A)** Intraoperative exploration showed that an 18*15 cm cystic mass(black asterisk) was seen in the right ovary. **(B)** The metal beads(black arrow) and spring(white arrow) were eliminated through the colostomy. **(C)** The wall of the weighted tip(white arrow) was damaged caused by the biopsy forceps. **(D)** A knot was tied at the tip of the ileus tube before insertion (“thread-knotting” method). **(E)** The ileus tube could be inserted easily by grasping the thread (black arrow) with biopsy forceps (white arrow).

According to the intraoperative exploration, an 18*15 cm cystic mass was seen in the right ovary (**Figure 3A**), and a 5*3 cm cystic mass was seen in the left ovary. At a distance of 20 cm from the ileocecum, the terminal ileum was infiltrated by and adhered to the right mass, the proximal small intestine was significantly dilated, and the left mass had infiltrated and adhered to the sigmoid colon. The uterus and cervix were not clearly distinguishable from the rectum. The tip of the ileus tube was located in the left lower abdomen.

The patient recovered well after the operation. The postoperative pathologic examination showed moderately differentiated adenocarcinoma with lymph node involvement (1/13) invading beyond the rectal wall; the patient was positive for intravascular tumor thrombus and nerve involvement; and the resection edge was negative. Adenocarcinoma tissue was found in the uterus and bilateral appendages. The immunohistochemical marker results were as follows: CK20 (+), SATB-2 (+), and PAX-8 (-) (suggesting that the tumor had originated from the rectum).

On April 9, 2022, CT did not show intestinal obstruction or intestinal leakage but showed separation between the metal beads at the head of the ileus tube (**Figure 2C**). When we removed the ileus tube, the metal beads and spring were not observed.

Considered that the patient had no signs of intestinal obstruction or perforation, she was given conservative treatment, including paraffin oil and a liquid diet. Paraffin oil was chosen to promote bowel motility, and the liquid diet was a diet that reduced stool formation and did not interfere with fecal observation, plus it did not affect the timing of emergency surgery in case of failure of conservative treatment.

Follow-up CT showed that six scattered metal balls had moved toward the distal small intestine, with no signs of intestinal obstruction or perforation (**Figures 2D, E**). On April 19, 2022, the metal beads and spring were eliminated through the colostomy (**Figure 3B**), CT showed no metallic shadow, and the other organs were not abnormal (**Figure 2B**). The patient received treatment with sindilizumab in combination with fruquintinib on May 6, 2022 and completed 18 cycles of the regimen. The patient was in good general condition. The metastatic nodules of both lungs were stable, and no pelvic metastatic lesions were found in the last follow-up. The timeline is shown in **Table 1**.

**Table 1:** The medical history of the patient.

Time series	Diagnosis and treatment details
September 20, 2016	The patient underwent radical proctectomy (DIXON) for rectal cancer, and received adjuvant therapy after surgery.
October 8, 2020	Imaging showed multiple metastatic soft masses located in the pelvic and pulmonary areas.
Between October 2020 and March 2022	The patient was treated with the chemotherapy plus targeted therapy.
In March, 2022	The patient presented with right lower abdominal pain because of tumor progression.
March 28,2022	An ileus tube was inserted because of symptoms of acute intestinal obstruction.
April 4, 2022	Bilateral ureteral catheterization was performed before surgery (to avoid a ureteral injury), extensive total hysterectomy + bilateral adnexal resection + rectal Hartmann operation + partial small bowel resection + intestinal adhesion release was performed.
April 9, 2022	When the ileus tube was removed, the metal beads and spring were not seen.
April 19, 2022	The metal beads and spring were eliminated through colostomy after conservative treatment.
From May 2022 to present	Targeted therapy and immunotherapy were continued, and the metastatic nodules of both lungs were stable, and no pelvic metastatic lesions were found in the last follow-up.

## Discussion

An ileus tube can approach or reach the proximal segment of the obstruction, has a large drainage volume and high drainage efficiency, and can play an active role in decompression (9). The guidelines point out that in the nonoperative treatment of adhesive intestinal obstruction, a long three-lumen nasointestinal tube is more effective than a nasogastric tube (it needs to be placed under endoscopy) (10, 11).

The reasons why the current patient underwent surgery are as follows: 1. Since the recurrent tumor nearly completely compressed the small bowel, decompression therapy failed to fundamentally improve the patient’s symptoms, and the patient’s nutritional status was too poor for the patient to tolerate the comprehensive treatment. 2. Based on physical examination and imaging, this patient had a high chance of having her pelvic tumor removed. 3. The patient was intolerant to decompression tubes and had a strong desire for surgery. Eventually, the patient resumed a normal diet after the operation, improved her physical fitness, and was able to successfully complete the follow-up treatment.

There were several reasons for the rupture of the weighted tip. First, we were used to guiding the insertion of the tube by directly clamping the weighted tip with forceps and ignoring the damage to the wall caused by the clamp (**Figure 3C**). Second, because the retention time was too long(15d), the original damaged rubber of the weighted tip could be further damaged by the corrosion of digestive juice.

At present, there is no uniform standard for the retention time of ileus tube. In our country, only one scholar has ever reported rupture of a nasogastric tube in China, and the nasogastric tube was retained for more than two weeks in his case. Wang found that the average retention time of ileus tubes in the successful treatment group for small bowel obstruction(SBO) was 15 days using propensity score matching (PSM) analysis (12). Therefore, since the patients could fail to benefit from an ileus tube after 15 days, we recommended that the retention time was not more than two weeks.

This case is enlightening due to the following: 1. Endoscopic-assisted insertion should be performed under intravenous anesthesia to minimize any patient discomfort and distress that may interfere with the insertion of ileus tubes. 2. Previously, we were used to guiding the insertion of the tube by directly clamping the weighted tip with biopsy forceps and ignoring the damage to the wall caused by the clamp (**Figure 3C**). Now, we can tie a knot at the tip of the tube and then complete the insertion by tugging on the thread (the “thread-knotting” method) (**Figures 3D, E**). Ten patients with intestinal obstruction in our institution treated by ileus tube from March 2022 to October 2023 were successfully inserted with “thread-knotting” method. Informed consent for the procedures was obtained from all patients. In addition,our “thread-knotting” method not only prevents rupture of the weighted tip but also avoids damage to the anterior balloon. Because of the close distance between the anterior balloon and the weighted tip, it is easy to accidentally break the balloon during tube insertion (**Figure 3D**). 3. Since this patient had undergone a terminal ileal resection, the reason for the longer retention time of the ileus tube was to reduce anastomotic leakage through effective decompression.

In addition to our “thread-knotting” method, Yamaguchi et al. reported that the transnasal endoscope was moved into the descending duodenum for insertion of the guidewire, and after the endoscope removal, the ileus tube was inserted into the duodenum through the guidewire (13). Guo et al. reported that the guidewire was placed 2 cm above the tip of the long tube so that it could be grasped easily by the biopsy forceps, and the scope and tube were passed through the pylorus and advanced as far as possible (14).

The main shortcoming of this report was the lack of information on the fractured tip excreted out of the body. Nevertheless, this case report has complete imaging data detailing the entire process relating to the tube, from its rupture in the small intestine to its complete expulsion.

Although this woman is very lucky and has a good prognosis, her success does not rule out that other patients may need a secondary surgery due to obstruction or perforation caused by broken tips or metal beads. The purpose of this case report is to encourage physicians to be more vigilant to reduce the incidence of such adverse events.

## Conclusion

The procedure of endoscopy-assisted ileus tube insertion could be improved to reduce the incidence of tube-related rupture with our “thread-knotting” method and intravenous anesthesia.

## Data availability statement

The original contributions presented in the study are included in the article/supplementary material. Further inquiries can be directed to the corresponding author.

## Ethics statement

Written informed consent was obtained from the individual(s) for the publication of any potentially identifiable images or data included in this article.

## Author contributions

JM: Investigation, Project administration, Software, Supervision, Writing – original draft. YJ: Data curation, Writing – review & editing. CPZ: Formal analysis, Writing – review & editing. DW: Writing – review & editing. CXZ: Writing – review & editing. YZ: Writing – review & editing.
